# Ce-radical Scavenger-Based Perfluorosulfonic Acid Aquivion^®^ Membrane for Pressurised PEM Electrolysers

**DOI:** 10.3390/polym15193906

**Published:** 2023-09-27

**Authors:** Stefania Siracusano, Fausta Giacobello, Stefano Tonella, Claudio Oldani, Antonino S. Aricò

**Affiliations:** 1 CNR-ITAE, Institute of Advanced Energy Technologies, National Research Council, Via Salita S. Lucia Sopra Contesse 5, 98126 Messina, Italy; giacobello@itae.cnr.it (F.G.); arico@itae.cnr.it (A.S.A.); 2 Solvay Specialty Polymers, Viale Lombardia 20, 20021 Bollate (MI), Italy; stefano.tonella@solvay.com (S.T.); claudio.oldani@solvay.com (C.O.)

**Keywords:** water electrolysis, hydrogen, polymer electrolyte membrane, radical scavenger, gas crossover, Aquivion^®^ membrane

## Abstract

A Ce-radical scavenger-based perfluorosulfonic acid (PFSA) Aquivion^®^ membrane (C98 05S-RSP) was developed and assessed for polymer electrolyte membrane (PEM) electrolyser applications. The membrane, produced by Solvay Specialty Polymers, had an equivalent weight (EW) of 980 g/eq and a thickness of 50 μm to reduce ohmic losses at a high current density. The electrochemical properties and gas crossover through the membrane were evaluated upon the formation of a membrane-electrode assembly (MEA) in a range of temperatures between 30 and 90 °C and at various differential pressures (ambient, 10 and 20 bars). Bare extruded (E98 05S) and reinforced (R98 05S) PFSA Aquivion^®^ membranes with similar EWs and thicknesses were assessed for comparison in terms of their performance, stability and hydrogen crossover under the same operating conditions. The method used for the membrane manufacturing significantly influenced the interfacial properties, with the electrodes affecting the polarisation resistance and H2 permeation in the oxygen stream, as well as the degradation rate, as observed in the durability studies.

## 1. Introduction

In recent years, the interest towards renewable energies has enormously grown because of their strategic role in the future energy system and the fight against climate change and global warming [1,2,3,4].

In this regard, green hydrogen can become an effective energy vector. Green hydrogen production, by means of water electrolysis fed by photovoltaic plants and wind turbines, is the most promising technology in terms of reliability and sustainability [5,6,7,8]. Green hydrogen has already received significant attention since it can contribute to reducing the emissions of segments that are difficult to decarbonise, also offering a valid option for the storage of renewable electricity [9].

Several electrolysis processes have been developed for water splitting, such as alkaline electrolysers, solid oxide systems and anion (AEM) and proton exchange (PEM) membrane electrolyses [10,11]. 

Among all electrolysis technologies, PEM electrolysers can work at a much higher current density, e.g., 4 A cm^−2^ and at cutting-edge efficiency, thus saving capital costs and energy consumption [12,13,14,15,16,17,18]. Moreover, PEM electrolysis can operate in high-pressure conditions in order to reduce the downstream mechanical gas compression when renewable H_2_ needs to be stored, leading to higher efficiency, lower costs and increased safety levels [19,20]. However, the cost of the hydrogen produced by PEM electrolysis is still expensive compared to centralised natural gas reforming, and a large reduction in capital costs is needed for an extensive deployment in conjunction with a reduced cost of renewable electricity [21]. Some strategies are currently used to decrease capital costs regarding operations at high production rates (high current density) by minimising the quantities of precious metals used as catalysts for oxygen and hydrogen evolution reactions and the replacement of most of the titanium in bipolar plates with coated stainless steel [22,23,24]. Operation at higher current density values would lead to an increasing rate of hydrogen production according to the Faraday law. Nevertheless, a high current density corresponds to a high ohmic loss with a consequent efficiency decrease. This drawback can be overcome by using thinner membranes, especially if these can operate in the presence of a recombination catalyst [25]. A reduction of the membrane thickness can lead to a decrease in resistance; however, in parallel, it can produce an increase in the hydrogen concentration in the anodic compartment [25]. If the cell operates under differential pressure conditions and high temperatures, a thin membrane can cause a large rise in the gas crossover.

An increase in the hydrogen concentration in the O_2_ stream can lead to significant safety problems if the flammability limit (4% vol H_2_ in O_2_ at room temperature and pressure) is surpassed [26]. It is, therefore, necessary to mediate between the decrease of the membrane thickness, which brings to better performance, and the effects on hydrogen crossover in the oxygen stream at the anode.

Short-side-chain PFSA Aquivion^®^ membranes are featured by their good ionic conductivity and low gas crossover due to higher crystallinity and lower equivalent weight with respect to the longer side-chain perfluorosulfonic acid membranes benchmark (Nafion^®^) [27,28]. Their high glass transition temperature allows these short-side-chain membranes to operate in a wide range of temperatures [29,30]. In order to reduce the hydrogen crossover and improve the stability, Pt nanoparticles, nanofibrous reinforcements or specific nanofillers can be integrated into the membrane structure [31,32,33,34,35,36,37,38]. 

Radical scavengers, based on Ce or Mn as metal or oxide nanoparticles, have been observed to protect the membrane in fuel cells, reducing the degradation rate [39,40,41,42]. Formation of hydroxyl radicals (HO•) in the proton exchange membrane (PEM) upon gas crossover leads to the breaking of the ionomer chain and, thus, to the degradation of the membrane. Radical scavengers show a faster reaction rate with the formed hydroxyl radicals than the reaction rate of peroxyl radicals with the polymer membrane. A radical scavenging mechanism of cerium (IV) oxide is reported in Ref. [41].

Therefore, radical scavengers can mitigate the polymer chemical degradation due to gas crossover [43]. To our knowledge, no relevant studies have been carried out on the use of radical scavengers in PEM electrolysis. The present work aims to cover such a gap.

In light of these considerations, the present work shows how the properties of a thin short-side-chain Aquivion^®^ membrane with a thickness of 50 µm, containing Ce-radical scavenger powder (C98-05S-RSP), assessed in a differential pressure PEM electrolyser, can considerably affect the performance and stability with respect to bare Aquivion^®^ membranes, extruded and reinforced (E98 05S and R98 05S, respectively), as previously investigated [18,44]. 

The electrochemical properties and gas crossover of the membrane under study were evaluated in a range of temperatures between 30 and 90 °C and at various differential pressure (pressurised H_2_; non-pressurised O_2_) values (ambient, 10 and 20 bars). The electrocatalytic layers, which were deposited onto the membrane to form the MEA, consisted of Pt/C for the cathode and IrRuO_x_ for the anode in combination with Aquivion^®^ ionomer dispersion to enhance the electronic percolation within the catalytic layers.

It is observed that the method used for membrane manufacturing significantly influences the properties at the interface with the electrodes in terms of the polarisation resistance and degradation rate in durability studies.

## 2. Experimental Section

### 2.1. Membrane-Electrode Assemblies (MEAs) Preparation and Characterization

Bare E98-05S-extruded and R98-05S-reinforced membranes were previously studied [18], whereas a recast Ce-radical scavenger-based Aquivion^®^ membrane was developed here. All membranes were formed with a thickness of 50 µm.

Specifically, starting from the same polymer, Aquivion^®^ PerfluoroSulfonic Acid (PFSA), three different grades of membranes were prepared by Solvay Specialty Polymers, namely extruded, reinforced and cast ones. The main specifics of these membranes are reported in Table 1.

The extruded membrane was obtained from a melt extrusion process from a precursor resin (in –SO_2_F form) and then transformed into the final, conductive –SO_3_H form. The melt extrusion process typically produces anisotropic membranes characterised by polymer chain orientation in the machine direction [45]. These membranes show different mechanical behaviours and dimensional changes in transversal and machine directions upon soaking in hot water. 

The reinforced membrane with ePTFE (expanded PolyTetraFluoroEthylene) support was produced by impregnation of a support with the Aquivion^®^ dispersion in –SO_3_H form. The support is used to increase the mechanical strength of the membrane while maintaining a constant electrochemical performance and transport properties. 

The Ce-scavenger-based cast membrane (Cast with RSP) was produced by spreading the ionomer dispersion in –SO_3_H form onto an inert support (PTFE/glass fibres or polyimide liners in industrial lines or tempered glass in the laboratory) followed by solvent removal and membrane consolidation at a high temperature. The Ce-scavenger supported on sulfonated silica was prepared and introduced in the membrane, as reported in Ref [46].

Briefly, the synthesis used was a wet impregnation technique. The ratio among the Si and Ce precursors and ammonium sulphite was 8:1.5:0.5. These were suspended in water, and after undergoing a thermal treatment of 300 °C, a powder was obtained, which had good dispersion properties for Ce and the correct anchoring of sulphur groups on the silica surface [46].

Typically, cast membranes are isotropic, showing the same behaviour in both machine and transversal directions. 

The Aquivion^®^ ionomer dispersion (D98-06AS, EW: 980 g/mol, 6 wt% solid content end-capped with -CF_3_ groups) that was used to promote bonding between the catalyst layer and the membrane was also provided by Solvay Specialty Polymers.

**Figure 1:**
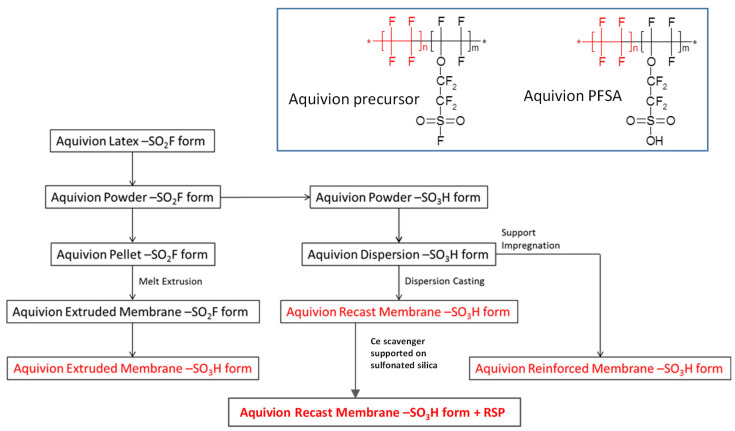
Flowchart of Aquivion^®^ proton exchange membranes preparation. Inset: chemical structure of Aquivion^®^ precursor (**left**) and its final form (**right**).

A main difference among the different preparations relies on the fact that extruded membranes are manufactured from a resin in its precursor –SO_2_F form (Figure 1, inset). Thereafter, the obtained film is hydrolysed to the –SO_3_H form (Figure 1, inset), whereas the other grades, being prepared from Aquivion dispersions in the –SO_3_H form, are already in the final, conductive form, and further treatment is not required. 

The three different grades of membranes had a thickness of 50 µm and were produced with a polymer having an EW (equivalent weight) of 980 g/mol.

A polymer powder in –SO_2_F form was melt-processed to pellets (feed for film extruder), hydrolysed and then dispersed in water to obtain the Aquivion^®^ dispersions. The membrane and ionomer dispersion production processes are shown in Figure 1. 

A catalyst-coated membrane (CCM) method was employed for the preparation of membrane–electrode assemblies [47]. The catalyst loading was 0.1 Pt and 0.37 Ir/Ru mg cm^−2^ at the cathode and anode, respectively. A Pt/C catalyst was used at the cathode, whereas the anode was IrRuOx (Ir:Ru = 70:30).

The Ce-scavenger-based Aquivion^®^ membrane, named C98 05S-RSP, with an equivalent weight of 980 g/mol and a thickness of 50 µm, was treated with sulfuric acid (0.5 mol L^−1^) and distilled water to obtain a purified product for use as the electrode separator in the PEM electrolytic cell. The bare membranes were treated as reported in a previous paper [18] before their use.

The MEA manufacturing involved the use of an alcoholic ink consisting of catalysts developed and reported in previous papers [48,49], and the Solvay ionomer was deposited by means of a spray deposition technique. The anodic and cathodic catalyst–ionomer inks were prepared by mixing the respective catalyst with the ionomer in the defined ratios, as shown in Table 2, and treated with ethanol in an ultrasonic bath. 

The obtained dispersions were sprayed directly on both sides of the membrane located on a heating metal plate at T = 80 °C. The catalyst loadings of the investigated MEAs are also reported in Table 2. CCMs were, lastly, hot-pressed at T = 190 °C while applying a force of 3 kN for 2 min in order to promote the adhesion of the catalytic layers on the membrane. The heat treatment was chosen on the basis of the glass transition temperature of the Aquivion^®^ polymer [50]. A carbon cloth (GDL ELAT from ETEK) and a titanium fibre mesh (Bekaert Toko Metal Fiber Co., Zwevegem, Belgium) featuring a 78% porosity, 20 µm fibre diameter and 0.3 mm thickness were used as gas diffusion layers (GDLs) for the cathode and anode, respectively. 

### 2.2. Electrochemical Investigations

The performance and stability of the MEAs under study were investigated at different temperatures, in a range of 30–90 °C, and at different pressure conditions (ambient, 10 and 20 bars). The water flow rate was 1 mL∙min^−1^cm^−2^. 

**Figure 2:**
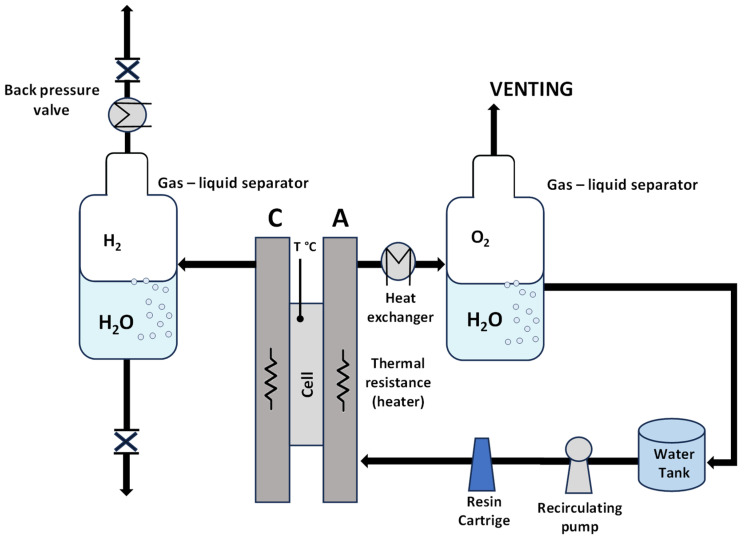
Scheme of the experimental setup.

A preliminary screening of the prepared MEAs at ambient pressure was carried out in an electrolysis cell with an active area of 5 cm^2^, equipped with titanium and graphite plates at the anode and cathode compartments, respectively. A circular-shaped single cell with an active area of 8 cm^2^ was used for the high-pressure measurements. Deionized water (Milli-Q integral Millipore, Burlington, MA, USA) with a resistivity of 18.2 mΩ cm was allowed to flow and recirculate only in the anode compartment for both systems with different active areas and different pressure conditions. A scheme of the experimental setup is reported in Figure 2.

Electrochemical tests, such as the galvanostatic polarisation curves, were carried out by using a power supply of 42 A (Keithley, model: 2268-20-42 DC Power Supply, Cleveland, OH, USA). The polarisation curves were carried out until a cell voltage of 2.2 V was achieved. An Autolab Metrohm potentiostat/galvanostat, equipped with a 20 A current booster and a frequency response analyser (FRA), was used to perform the impedance spectroscopy studies (EIS). As it is well known, the impedance is expressed by the following equation:

(1) $$\frac{\dot{V}}{\dot{I}}=\dot{Z}=Z^{\prime }+j\;Z^{\prime \prime }$$

where $\dot{Z}$ is the phasor of the impedance, $\dot{V}$ is the phasor of the cell potential, $\dot{I}$ is the phasor of the current intensity, $Z^{\prime }$ is the real part of the impedance and $Z^{\prime \prime }$ is the imaginary part of the impedance [51].

These were conducted at 1.5 V and 1.8 V by varying the oscillation (10 mV) frequency from 100 kHz to 100 mHz in a single sine mode in order to determine the series and polarisation resistances, *R_s_* and *R_p_*, respectively. 

The pressurised 8 cm^2^ single-cell housing served to determine the hydrogen concentration present in the anodic stream under various differential pressures using specific analytic tools.

The quantitative analysis of the hydrogen concentration was performed at a constant current density (2 h under steady-state conditions for each applied current density) by a pressurised cell setup in combination with a micro gas chromatograph (Varian Micro GC, SpectraLab, Markham, ON, Canada). The anodic gas stream was passed through a drying device before being analysed.

## 3. Results and Discussion

**Figure 3:**
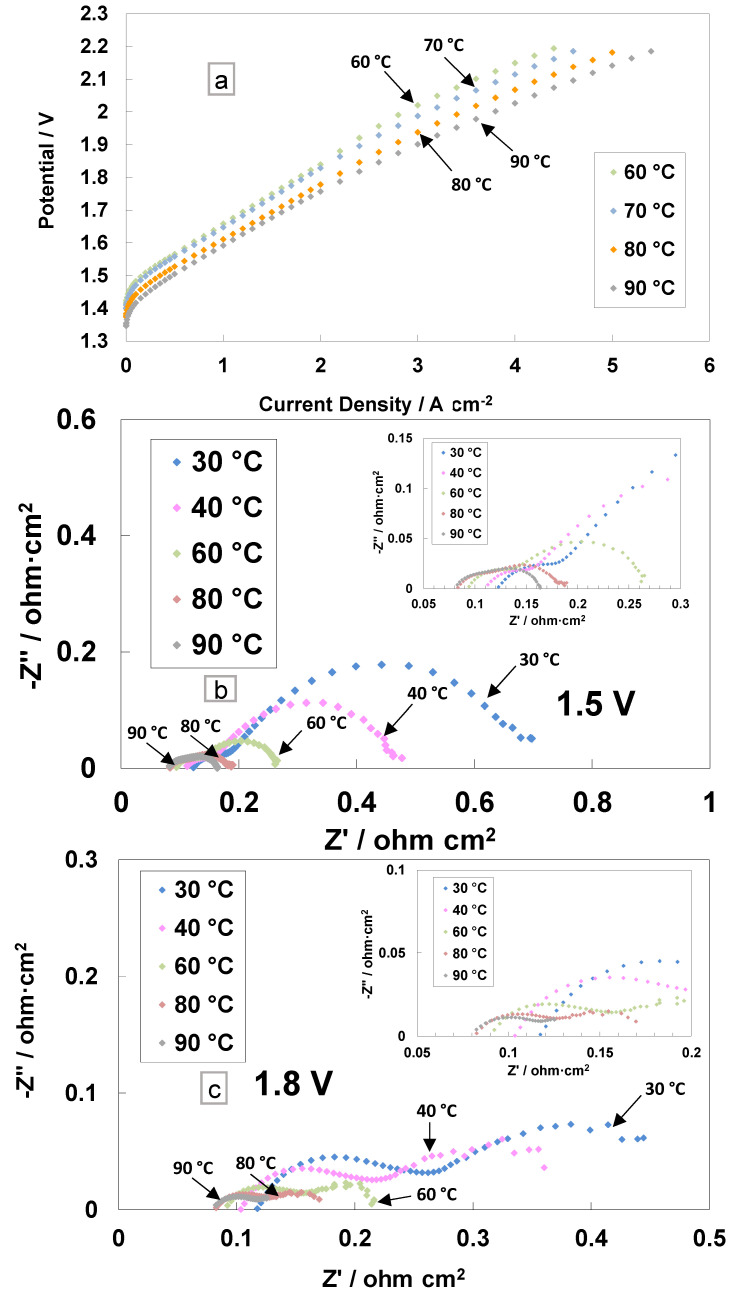
Polarisation curves (**a**) and AC-impedance spectra at 1.5 V (**b**) and 1.8 V (**c**) at different temperatures for the MEA based on cast membrane equipped with a Ce-radical scavenger.

The polarisation curves and electrochemical impedance analyses for the cast membrane containing the radical scavenger-based MEA are reported in Figure 3. The I–V measurements were performed in the electrolysis single cell at different temperatures (from 60 to 90 °C) and ambient pressure (Figure 3a).

This range of temperatures is deemed suitable for the PEM electrolysis operation [52]. The current density increases, resulting in a reduction of the activation and ohmic losses with the increase in temperature. A current density of 4 A cm^−2^ at 2 V, with the cast membrane with the radical scavenger-based MEA, was achieved at 90 °C. A slope variation at high current densities was evident in the polarisation curves, probably due to an in situ modification of the membrane properties. In particular, the replacement of Ce ions from the Ce-SiO_2_-SO_3_H scavenger with protons (RSP) in the membrane sulphonates group may lead to a series of resistance reductions.

The AC-impedance analysis was carried out under constant voltage conditions of 1.5 V and 1.8 V (Figure 3b,c). These voltage values have been selected to analyse the behaviour of the MEA in the activation zone (1.5 V) and the ohmic zone (1.8 V). The electrochemical impedance spectra were recorded from 30 to 90 °C and reported in Nyquist plots. At low temperatures, the main impedance contribution is due to the polarisation resistance *R_p_* (electrodes’ contribution), whereas at high temperatures (80–90 °C), the series resistance *R_s_* (ohmic losses) dominates the polarisation behaviour (associated with the total resistance, *R_t_*) with respect to the polarisation resistance (Figure 3b,c and inset).

At 1.5 V, the series resistance, the high-frequency intercept on the real axis, decreased by increasing the temperature (Figure 3b). At 30 °C, *R_s_* is 0.118 Ohm cm^2^, whereas at 90 °C, the value decreased down to 0.082 Ohm cm^2^. This effect was even more evident for the polarisation resistance, i.e., the difference between the total resistance (low-frequency intercept on the real axis) and *R_s_*. At 30 °C, a value of 0.580 Ohm∙cm^2^ was recorded, whereas at 90 °C, the value decreased down to 0.081 Ohm∙cm^2^.

**Figure 4:**
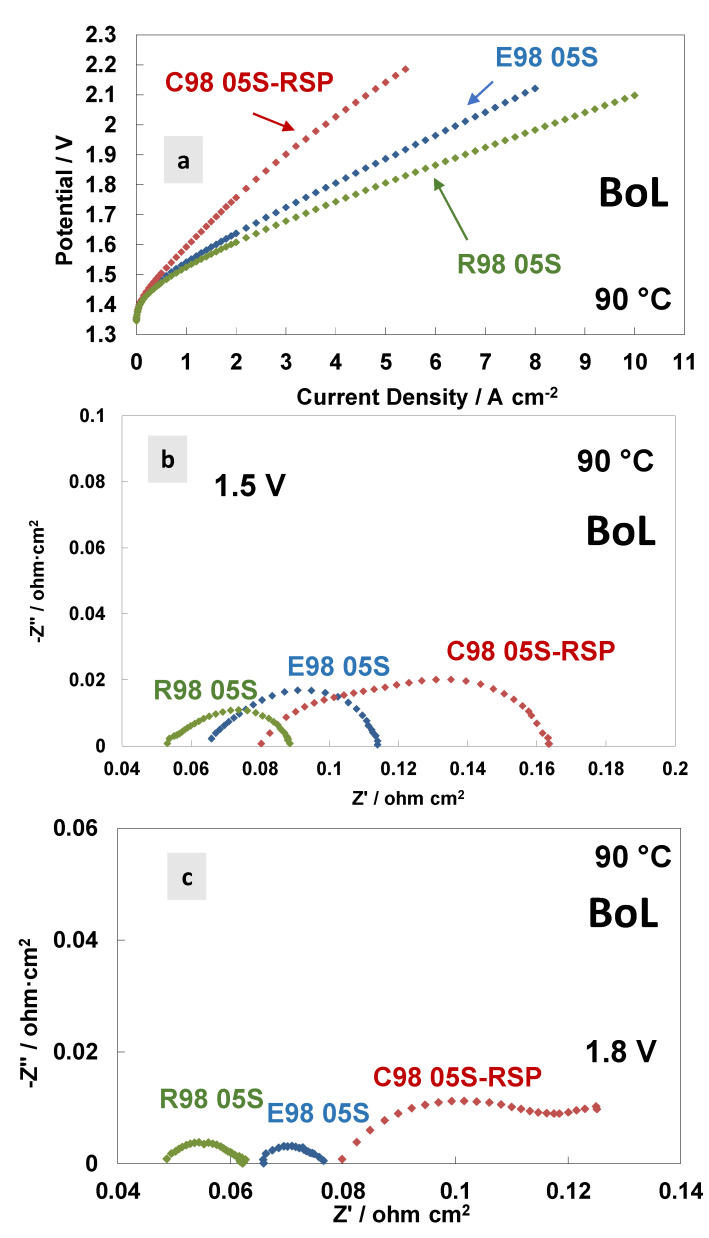
Polarisation curves (**a**) and AC-impedance spectra at 1.5 V (**b**) and 1.8 V (**c**) at 90 °C of the MEAs with extruded, reinforced and cast radical scavenger membranes at the beginning of life (BoL).

In Figure 4, a comparison of the polarisation curves and AC-impedance spectra at 90 °C at the beginning of life (BoL) of the MEAs is reported based on the different membranes. Figure 4a shows the polarisation curves carried out at 90 °C. A similar activation behaviour is quite evident at 90 °C for all the MEAs tested. This means that the anodic catalyst properties—the main ones responsible for the activation process (the oxygen evolution reaction is the rate-determining step in this process [53])—are independent of the membranes used. At high current densities (ohmic and diffusional contributions), the polarisation curves reveal higher cell voltages for the C98 05S-RSP membrane-based MEA, showing a much higher slope with respect to the extruded and reinforced membrane-based MEAs. The latter holds the best performance. At 4 A cm^−2^, the potential value of the MEA that is based on C98 05S-RSP has an overpotential of 280 and 220 mV higher when compared to R98 05S- and E98 05S-based MEAs, respectively.

The AC-impedance spectra of the MEAs based on the different membranes, acquired at 1.5 V and 1.8 V, respectively, are reported in Figure 4b,c. All membranes are based on the same ionomer and have the same thickness; however, the different resistance values (*R_s_*, *R_p_* and *R_t_*) for the three MEAs tested are evident in the spectra, with an increase in the resistance passing from the reinforced to extruded and cast based-MEAs. This evidence is probably due to the different reaction rates for the membranes in the spectra recorded at 1.5 V and 1.8 V (Table 3 and Table 4). A higher current density is recorded during the impedance spectra at fixed potentials when a lower resistance is observed. At 1.8 V (Figure 4c), in the case of the C98 05S-RSP membrane-based MEA, some additional low-frequency contributions appear, as testified by the presence of a (second) semicircle. It is probably a diffusion-related problem due to a blockage of the functional groups by the Ce ions, which delays the proton transport.

Usually, in the ohmic zone, the contribution of the series resistance is prevailing compared to the polarisation resistance, considerably affecting the achievable current density in the specific voltage range [18,44]. The cast membrane-based MEA with radical scavengers showed a significantly high polarisation resistance at different temperatures except at 90 °C (Figure 3c). The high *R_p_* was responsible for the relatively modest initial performance (Figure 4a), whereas diffusional problems with a possible lack of electronic percolation were due to a non-optimal electrode–electrolyte interface. The *R_s_* and *R_p_* values at 1.8 V, achieved at 90 °C, were 0.082 Ohm cm^2^ and 0.040 Ohm cm^2^, respectively.

**Figure 5:**
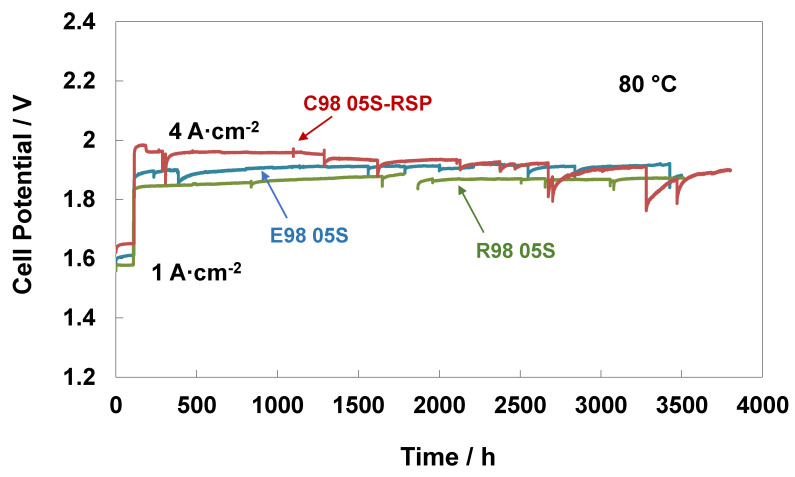
Steady-state durability test at 1 and 4 A∙cm^−2^ and 80 °C of the MEAs with extruded, reinforced, and cast radical scavenger-based membranes.

In addition to performance, stability features are crucial for electrolysis systems, especially when using low catalyst loadings and working at high current densities as fundamental factors for reducing CAPEX [54,55]. Steady-state durability tests of 3500 h at 80 °C and ambient pressure were carried out for the extruded, reinforced and cast with RSP membrane-based MEAs. Figure 5 reports the durability tests with an initial conditioning step at 1 A∙cm^−2^ for about 100 h and subsequently at 4 A cm^−2^ for about 3500 h. A higher initial increase in cell potential at both current densities (1 and 4 Acm^−2^) was observed for the C98 05S-RSP-based MEA, with a loss of performance of about 140 mV (the recorded value after the 150 h test) compared to the bare membranes. After 150 h, the MEA based on the cast membrane increased performance and progressively reduced the gap versus the bare membranes. This is possibly associated with the replacement of Ce ions with protons in the sulphonate groups occurring during the steady-state test for the C98 05S-RSP membrane. This improvement could be due either to a reduction of Ce ions over time or a rearrangement of the interaction between the membrane and scavenger. At the end of the test, a recovery rate of about 22 µV/h was observed for the cast-based MEA as evaluated by a best linear fitting procedure. Because of some maintenance work on the test station, an interruption of about 100 h was necessary for the test regarding the R98-05S-based MEA. This was needed to replace the ion exchange resin cartridge since the water conductivity was increasing beyond the requested quality limit (18 MOhm). The MEA behaviour after this maintenance work was slightly better. At the end of the steady-state durability tests of 3500 h, the C98 05S-RSP-based MEA improved its performance, and E98 05S worsened it, whereas the R98 05S cell potential appeared more stable than that of C98 05S-RSP; at 3500 h, both R98 05S and C98 05S-RSP had a similar performance. The MEAs based on E98 05S and R98 05S showed degradation rates of 21 μV/h and 13 μV/h, respectively. 

Of course, during a 3500 h durability test, interruptions are sometimes occurring as a consequence of laboratory and test station maintenance. According to our experience, these interruptions usually affect the recovery of reversible losses in a short time span; however, they do not modify the macroscopic behaviour of the functional cell components during a large time window. This is also the reason why we have considered an extensive durability test of 3500 h instead of short tests; the main durability characteristics of such an extensive timespan are much less affected by random interruptions. 

**Figure 6:**
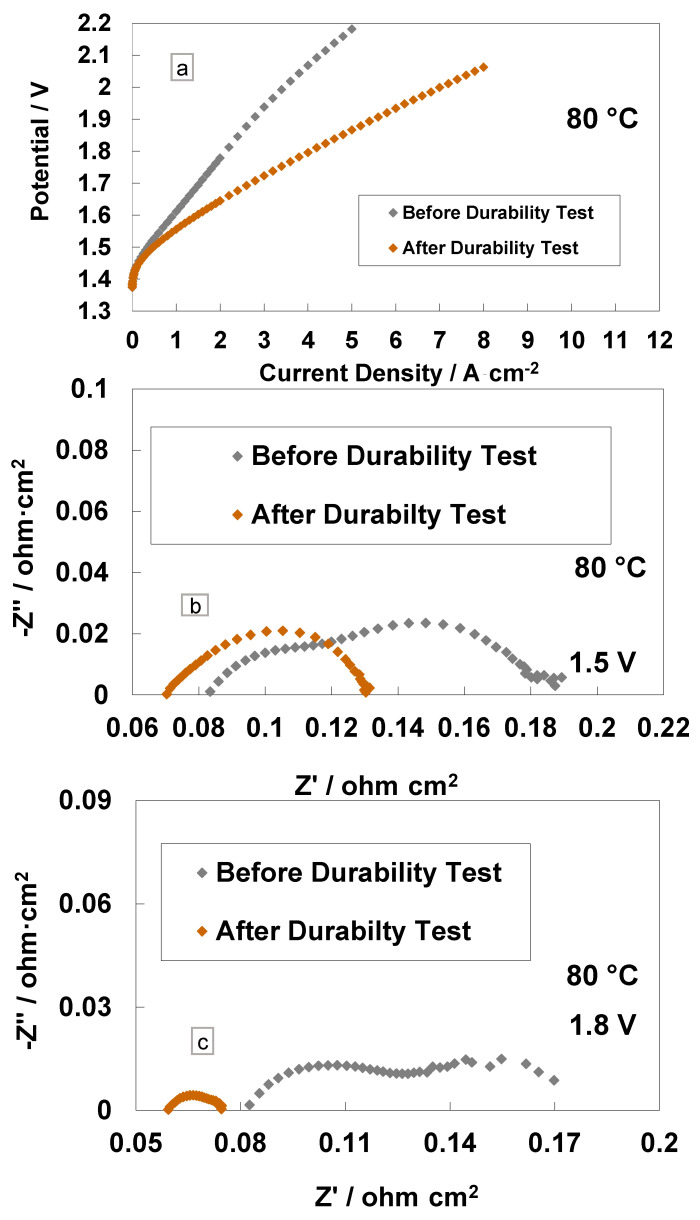
Polarisation curves (**a**), AC-impedance spectra at 1.5 V (**b**) and AC-impedance spectra at 1.8 V and 80 °C (**c**) before and after steady-state durability test for the MEAs with cast membrane containing a Ce-radical scavenger.

A comparison of the polarisation curves and impedance spectra for the cast membrane with the RSP-based MEA at the BoL and after the steady-state operation (3800 h) is reported in Figure 6. It is interesting to see a large increase in performance in terms of reduced cell potential at the same current density (Figure 6a). At 4 A cm^−2^ and 80 °C, the cell based on the Ce-containing membrane reached the potential value of 1.79 V, whereas, at the BoL, it was close to 2.1 V, i.e., the overpotential was higher than 270 mV at the BoL (590 mV at BoL vs. 320 mV at EoL) versus the thermoneutral potential (1.48 V) at the same current density and temperature. In particular, at 4 A∙cm^−2^, the voltage efficiencies, with respect to the high heating value (HHV), were 72% and 82% before and after the 3800 h steady-state test, respectively, for the Ce-based membrane.

The impedance spectra, recorded before and after the durability tests at 1.5 V and 1.8 V, are reported in Figure 6b,c. A decrease of *R_s_* and *R_p_* after 3500 h of operation is evident. The slight reduction of *R_s_* after the durability test is due to a slight cleaning and/or thinning of the membrane over time [18,44]. More evident is the decrease of *R_p_* after the 1.5 V and 1.8 V tests with a reduction in the mass transport contribution. This is probably due to a modification of the interface or to the rearrangement of the Ce-based scavenger (RSP) contained in the membrane in the first period of MEA life. This is quite unusual in this field. It relates to such a novel configuration for the membrane, which contains a compound designed to improve its durability by reducing the formation of the peroxyl radicals occurring by the effect of gas crossover.

**Figure 7:**
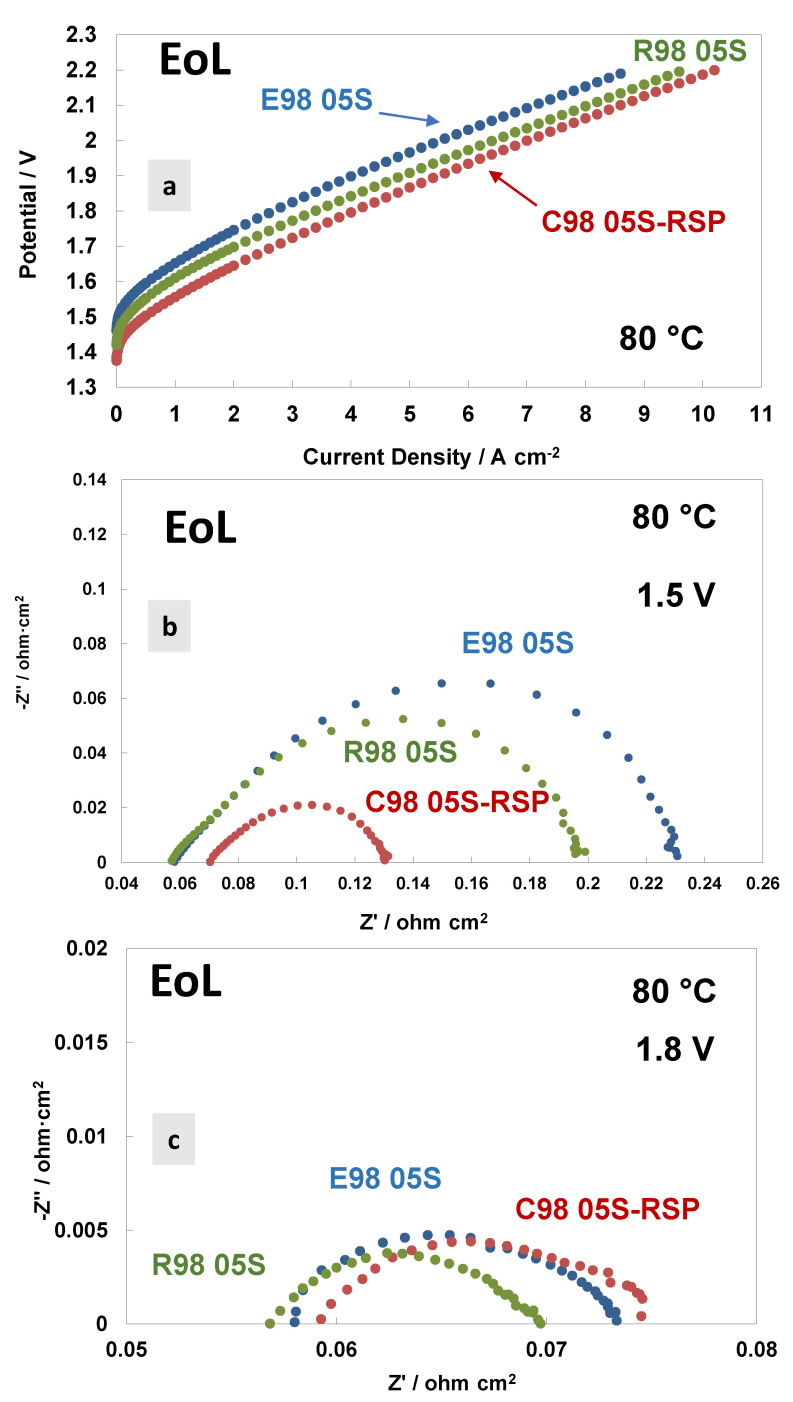
Polarisation curves (**a**) and AC-impedance spectra at 1.5 V (**b**) and 1.8 V (**c**) at 80 °C of the MEAs with extruded, reinforced and cast radical scavenger membranes at the end of life (EoL).

A comparison of the polarisation curves and AC-impedance spectra at 80 °C and at the end of life (EoL) of the MEAs based on the different membranes are reported in Figure 7. Figure 7a shows the polarisation curves carried out at 80 °C. Interestingly, the Ce-based membranes show a much better performance and lower polarisation resistance than bare membranes. Thus, the Ce-based membrane improved in performance, while the bare membranes degraded in performance with time. A lower cell voltage onset in the polarisation curve is quite evident for the C98 05S-RSP-based MEA. This is mainly due to a decrease in polarisation resistance at a low frequency (Figure 7b). The radical scavenger-based membrane shows, therefore, a kinetic improvement over the bare membranes during the durability test. This means that there is a competitive advantage in using this membrane over the bare membranes after an initial reduction period of 3000 h. Considering that these MEAs have the same catalyst at both the anode and cathode, the difference is due to the manufacturing methodology of the membranes and their interfacial properties with the catalysts. It is clear that the C98 05S-RSP-based MEA improved the performance after the steady-state durability test compared to the bare membrane-based MEAs. At a high current density, the slope of the reinforced-based MEA is lower compared to the cast membrane-based MEA. This is due to a slightly higher ohmic resistance that remains in the C98 05S-RSP membrane (Figure 7b,c) after the stability test. 

**Figure 8:**
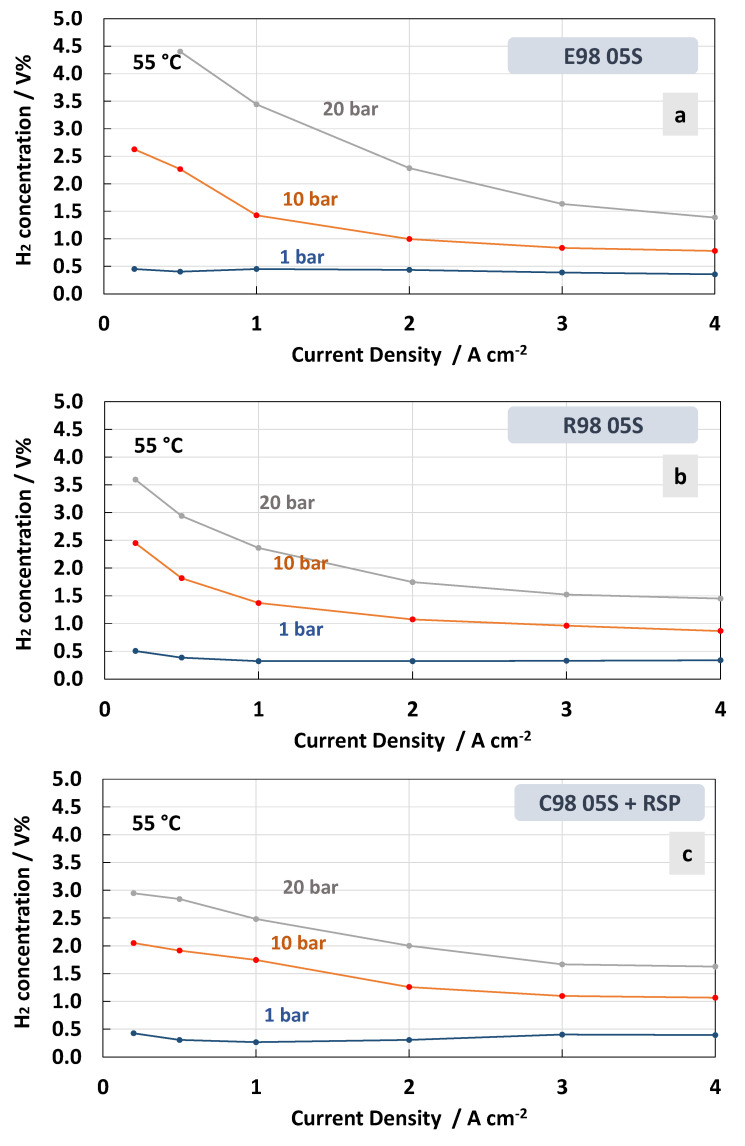
H_2_ concentration in O_2_ at 55 °C and 1, 10 and 20 bar differential pressure for the MEAs with extruded (**a**), reinforced (**b**) and cast containing Ce-radical scavenger (**c**) membranes.

**Figure 9:**
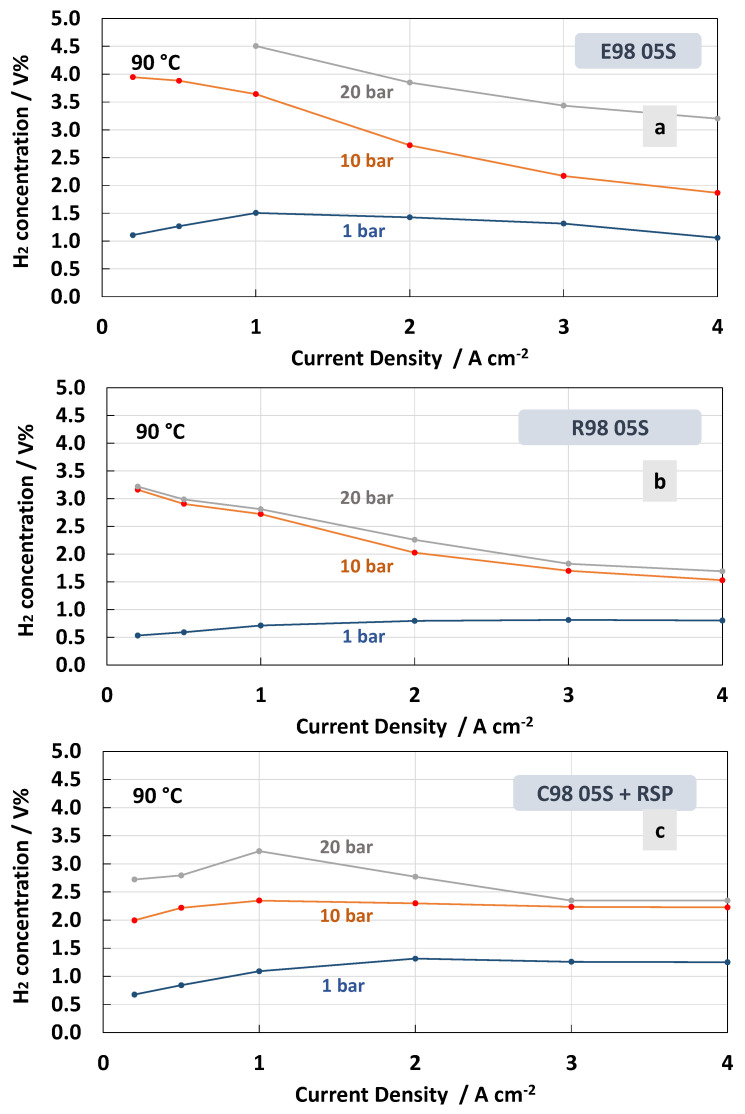
H_2_ concentration in O_2_ at 90 °C and 1, 10 and 20 bar differential pressure for the MEAs with extruded (**a**), reinforced (**b**) and cast radical scavenger (**c**) membranes.

The measurements of hydrogen concentration in the oxygen stream as a function of the current density at constant temperatures of 55 °C and 90 °C under differential pressure conditions are reported in Figure 8 and Figure 9. These represent two extreme conditions for the operating temperature, thus allowing us to obtain a more complete picture of the membrane’s behaviour. Thin membranes can cause a high hydrogen crossover in the oxygen under strength differential pressure [26,56,57]. The tests carried out for the MEAs with extruded, reinforced, and cast Ce-radical scavenger membranes at 55 °C and 1, 10 and 20 bar are reported in Figure 8. For all MEAs, at 1 bar (no pressurised O_2_; no pressurised H_2_), the amount of H_2_ concentration remained constant below 0.5 in the total range of the current densities, 0.2–4 A∙cm^−2^, whereas at 10 and 20 bar (no pressurised O_2_; pressurised H_2_) an increase in hydrogen concentration with a decrease in current density was observed (Figure 8a–c). This is due to the decrease in oxygen production with the lowering of the current density. Thus, the permeated H_2_ occurring as a consequence of the diffusion driven by a concentration gradient increases its relative content in O_2_. 

Therefore, when a differential pressure is applied, the hydrogen concentration in the oxygen stream increases at a very low current density. The lowest crossover was recorded for the MEA based on the cast membrane with the Ce-radical scavenger (Figure 8c). The value of the H_2_ concentration in the O_2_ stream remained below 2% in all the ranges of current densities and below 3% when 10 and 20 bars of differential pressure, respectively, were applied. This was probably due to a tortuosity effect of the Ce nanofiller in this membrane (cast with radical scavenger, C98 05S-RSP) compared to the extruded and reinforced membranes. It is interesting to observe that for the MEAs based on R98 05S and C98 05S-RSP membranes, the system can work below 2% of H_2_ in O_2_ at 10 bar differential pressure in the range of a current density of 0.5–4 Acm^−2^ (Figure 8b,c). This allows it to operate with a low partial load (15%).

The H_2_ concentration in O_2_ for the different membranes recorded at 90 °C is reported in Figure 9. At high temperatures, the MEA that was based on the E98 05S membrane showed a higher crossover in all ranges of the current densities and differential pressures (Figure 9a) compared to the measurements at 55 °C (Figure 8a). Furthermore, for the MEA based on the R98 05S membrane, a slight increase of H_2_ concentration in O_2_ was evident at all differential pressures, though at a low current density at 20 bar, the increase was less evident (3.2 H_2_ conc.% at 90 °C vs. 3.6 H_2_ conc.% at 55 °C) (Figure 9b). A similar situation occurred for the MEA equipped with C98 05S-RSP, where 2.6 H_2_ conc.% was recorded at 20 bar and 90 °C vs. 2.9 H_2_ conc.% at 55 °C and a low current density. These unusual phenomena may be related to the formation of water vapour at 90 °C in the non-pressurised anode compartment. This water vapour can generate a counter pressure counteracting the H_2_ crossover.

In general, low current density is a critical condition when operating in PEM electrolysis at high pressures. In the case of the MEAs based on the C98 05S-RSP membrane, in all ranges of current density, including 0.2 A cm^−2^ (5% partial load), the system is able to work well below the flammability limit [26,56,57].

Regarding the effect of the temperature, it is more difficult to derive a concluding better behaviour for the C98 05S-RSP compared to the R98 05S membrane. Probably, in both cases, the membrane behaviour as a function of temperature is positively affected by the presence of a filler or a reinforcement. It is also expected that other intrinsic properties of the polymer, such as the low equivalent weight and the high glass transition temperature, may play a role. These are essentially similar in all membranes. However, the extruded membrane shows a lower capability to mitigate the hydrogen crossover as a function of the temperature. 

## 4. Conclusions

A membrane-electrode assembly based on the chemically stabilised short-side-chain proton exchange Aquivion^®^ membrane containing a Ce-radical scavenger was investigated for operation at a high current density with a reduced concentration of H_2_ in O_2_ and with improved stability in a water electrolysis cell. However, at the beginning of life, the performances were low due to the exchange of protons with Ce ions from the radical scavenger, and it was observed that the performance improved with time, making this system more efficient than bare extruded and reinforced membranes. Moreover, a lower H_2_ concentration in O_2_ was observed for the Ce-scavenger-based membrane compared to the bare membrane-based MEAs. This indicates an increased tortuosity effect for the gas crossover induced by the Ce-based nanofiller.

The present study demonstrates that membrane manufacturing and the presence of a Ce nanofiller significantly influence the properties at the interface with the electrodes in a PEM water electrolysis cell. In particular, the Ce-based radical scavenger-based perfluorosulfonic acid Aquivion^®^ membrane provided excellent resilience to degradation and a lower H_2_ content in the oxygen. An increase in performance during a 3500 h durability test indicated that the nanofiller was effective in improving the system’s stability. Thus, the RSP integrated into Aquivion^®^ membranes represents a highly resistant and extremely performing alternative for highly safe and performing PEM electrolysers.

## Figures and Tables

**Table 1:** Main properties of the investigated membranes.

Membrane	Equivalent Weight (g/mol)	Thickness (µm)	Filler	Glass Transition Temperature Tg/°C	Conductivity at 80 °C and 100% RH (mS/cm)
**E 98 05S**	980	50	none	140	140.4
**C98 05S-RSP**	980	50	Ce-scavenger	140	131.3
**R98 05S**	980	50	ePTFE fibres	140	131.6

**Table 2:** Composition and catalyst loading of the investigated MEAs.

MEA	Anode		Membrane	Cathode		Ref.
	*IrRuOx Catalyst Loading* (mg_Ir+Ru_/cm^2^)	*Aquivion^®^ D98-06AS Ionomer* (wt.%)	*Aquivion^®^*	*Pt/C Catalyst Loading* (mg_Pt_/cm^2^)	*Aquivion^®^* *D98-06AS Ionomer* (wt.%)	
***Cast-RSP***	0.37	15	C98 05S-RSP	0.1	28	This work
***Extruded***	0.37	15	E98 05S	0.1	28	[18]
***Reinforced***	0.37	15	R98 05S	0.1	28	[18]

**Table 3:** Resistances and current density values recorded for the different membrane-based MEAs by AC-impedance spectra at 1.5 V and the I–V curve at 90 °C.

1.5 *V@*90 *°C*	*R_s_/mOhm cm* ^2^	*R_p_/mOhm cm* ^2^	*R_t_/mOhm cm* ^2^	*j/A cm* ^−2^
**R98 05S**	52	35	87	0.75
**E98 05S**	66	47	113	0.65
**C98 05S-RSP**	80	83	163	0.47

**Table 4:** Resistances and current density values recorded for the different membrane-based MEAs by AC-impedance spectra at 1.8 V and the I–V curve at 90 °C.

1.8 *V@*90 *°C*	*Rs/mOhm cm* ^2^	*Rp/mOhm cm* ^2^	*Rt/mOhm cm* ^2^	*j/A cm* ^−2^
**R98 05S**	49	13	62	4.9
**E98 05S**	66	10	76	3.9
**C98 05S-RSP**	80	40	120	2.3

## Data Availability

The data presented in this study are available on request from the corresponding author.

## References

[1] Olabi A.G., Abdelkareem M.A. (2022). Renewable energy and climate change. Renew. Sustain. Energy Rev..

[2] Yang Y., Javanroodi K., Nik V.M. (2022). Climate Change and Renewable Energy Generation in Europe—Long-Term Impact Assessment on Solar and Wind Energy Using High-Resolution Future Climate Data and Considering Climate Uncertainties. Energies.

[3] Pani A., Shirkole S.S., Mujumdar A.S. (2022). Importance of renewable energy in the fight against global climate change. Dry. Technol..

[4] Ge C., Sheng M., Yuan Y., Shi F., Yang Y., Zhao S., Wang J., Wang Z. (2023). Recent advances of the interfacial polymerization process in gas separation membranes fabrication. J. Membr. Sci..

[5] Calnan S., Bagacki R., Bao F., Dorbandt I., Kemppainen E., Schary C., Schlatmann R., Leonardi M., Lombardo S.A., Milazzo R.G. (2022). Development of Various Photovoltaic-Driven Water Electrolysis Technologies for Green Solar Hydrogen Generation. Sol. RRL.

[6] Ahmad H., Kamarudin S.K., Minggu L.J., Kassim M. (2015). Hydrogen from photo-catalytic water splitting process: A review. Renew. Sustain. Energy Rev..

[7] Nasser M., Megahed T.F., Ookawara S., Hassan H. (2022). Performance evaluation of PV panels/wind turbines hybrid system for green hydrogen generation and storage: Energy, exergy, economic, and enviroeconomic. Energy Convers. Manag..

[8] Ng K.H., Lai S.Y., Cheng C.K., Cheng Y.W., Chong C.C. (2021). Photocatalytic water splitting for solving energy crisis: Myth, Fact or Busted?. Chem. Eng. J..

[9] Capurso T., Stefanizzi M., Torresi M., Camporeale S.M. (2022). Perspective of the role of hydrogen in the 21st century energy transition. Energy Convers. Manag..

[10] Guo Y., Li G., Zhou J., Liu Y. (2019). Comparison between hydrogen production by alkaline water electrolysis and hydrogen production by PEM electrolysis. IOP Conf. Ser. Earth Environ. Sci..

[11] Hauch A., Küngas R., Blennow P., Hansen A.B., Hansen J.B., Mathiesen B.V., Mogensen M.B. (2020). Recent advances in solid oxide cell technology for electrolysis. Science.

[12] Immerz C., Schweins M., Trinke P., Bensmann B., Paidar M., Bystroň T., Bouzek K., Hanke-Rauschenbach R. (2018). Experimental characterization of inhomogeneity in current density and temperature distribution along a single-channel PEM water electrolysis cell. Electrochim. Acta.

[13] Chisholm G., Cronin L., Symes M.D. (2020). Decoupled electrolysis using a silicotungstic acid electron-coupled-proton buffer in a proton exchange membrane cell. Electrochim. Acta.

[14] Frensch S.H., Olesen A.C., Araya S.S., Kær S.K. (2018). Model-supported characterization of a PEM water electrolysis cell for the effect of compression. Electrochim. Acta.

[15] Olesen A.C., Frensch S.H., Kær S.K. (2019). Towards uniformly distributed heat, mass and charge: A flow field design study for high pressure and high current density operation of PEM electrolysis cells. Electrochim. Acta.

[16] Rost U., Wirkert F.J., Roth J., Brodmann M., Stiber S., Gago A.S., Friedrich K.A. (2022). A novel advanced test system for polymer electrolyte membrane water electrolysis based on hydraulic cell compression. Fuel Cells.

[17] Scheepers F., Stähler M., Stähler A., Rauls E., Müller M., Carmo M., Lehnert W. (2021). Temperature optimization for improving polymer electrolyte membrane-water electrolysis system efficiency. Appl. Energy.

[18] Siracusano S., Pantò F., Tonella S., Oldani C., Aricò A.S. (2022). Reinforced short-side-chain Aquivion^®^ membrane for proton exchange membrane water electrolysis. Int. J. Hydrog. Energy.

[19] Martin A., Trinke P., Stähler M., Stähler A., Scheepers F., Bensmann B., Carmo M., Lehnert W., Hanke-Rauschenbach R. (2022). The Effect of Cell Compression and Cathode Pressure on Hydrogen Crossover in PEM Water Electrolysis. J. Electrochem. Soc..

[20] Omrani R., Shabani B. (2021). Hydrogen crossover in proton exchange membrane electrolysers: The effect of current density, pressure, temperature, and compression. Electrochim. Acta.

[21] Salehmin M.N.I., Husaini T., Goh J., Sulong A.B. (2022). High-pressure PEM water electrolyser: A review on challenges and mitigation strategies towards green and low-cost hydrogen production. Energy Convers. Manag..

[22] Hubert M.A., King L.A., Jaramillo T.F. (2022). Evaluating the Case for Reduced Precious Metal Catalysts in Proton Exchange Membrane Electrolyzers. ACS Energy Lett..

[23] Rojas N., Sánchez-Molina M., Sevilla G., Amores E., Almandoz E., Esparza J., Cruz Vivas M.R., Colominas C. (2021). Coated stainless steels evaluation for bipolar plates in PEM water electrolysis conditions. Int. J. Hydrog. Energy.

[24] Lim T., Kim S.-K. (2022). Non-precious hydrogen evolution reaction catalysts: Stepping forward to practical polymer electrolyte membrane-based zero-gap water electrolyzers. Chem. Eng. J..

[25] Garbe S., Futter J., Schmidt T.J., Gubler L. (2021). Insight into elevated temperature and thin membrane application for high efficiency in polymer electrolyte water electrolysis. Electrochim. Acta.

[26] Ouimet R.J., Yu H., Mirshekari G., Zeng Z., Bonville L.J., Bliznakov S., Niedzwiecki A., Mani P., Capuano C., Ayers K.E. (2020). Development of Recombination Layers to Reduce Gas Crossover for Proton Exchange Membrane Water Electrolyzers By Reactive Spray Deposition Technology. ECS Meet. Abstr..

[27] Wu X., Scott K., Puthiyapura V. (2012). Polymer electrolyte membrane water electrolyser with Aquivion^®^ short side chain perfluorosulfonic acid ionomer binder in catalyst layers. Int. J. Hydrog. Energy.

[28] Shirvanian P., van Berkel F. (2020). Novel components in Proton Exchange Membrane (PEM) Water Electrolyzers (PEMWE): Status, challenges and future needs. A mini review. Electrochem. Commun..

[29] Ghielmi A., Vaccarono P., Troglia C., Arcella V. (2005). Proton exchange membranes based on the short-side-chain perfluorinated ionomer. J. Power Sources.

[30] Briguglio N., Pantò F., Siracusano S., Aricò A.S. (2020). Enhanced performance of a PtCo recombination catalyst for reducing the H2 concentration in the O2 stream of a PEM electrolysis cell in the presence of a thin membrane and a high differential pressure. Electrochim. Acta.

[31] Giancola S., Zatoń M., Reyes-Carmona Á., Dupont M., Donnadio A., Cavaliere S., Rozière J., Jones D.J. (2019). Composite short side chain PFSA membranes for PEM water electrolysis. J. Membr. Sci..

[32] Shin S.-H., Nur P.J., Kodir A., Kwak D.-H., Lee H., Shin D., Bae B. (2019). Improving the Mechanical Durability of Short-Side-Chain Perfluorinated Polymer Electrolyte Membranes by Annealing and Physical Reinforcement. ACS Omega.

[33] Chae K.-J., Kim K.-Y., Choi M.-J., Yang E., Kim I.S., Ren X., Lee M. (2014). Sulfonated polyether ether ketone (SPEEK)-based composite proton exchange membrane reinforced with nanofibers for microbial electrolysis cells. Chem. Eng. J..

[34] Lei J., Liu X., Chen X., Guan P., Feng W., Zhang J., Luo H., Liu F., Zhang Y. (2022). Multi-length-scale heterogeneous structured ion exchange membranes for cost-effective electrolysis and hydrogen production. Chem. Eng. J..

[35] Ballengee J.B., Pintauro P.N. (2013). Preparation of nanofiber composite proton-exchange membranes from dual fiber electrospun mats. J. Membr. Sci..

[36] Lee P.-C., Han T.-H., Kim D.O., Lee J.-H., Kang S.-J., Chung C.-H., Lee Y., Cho S.M., Choi H.-G., Kim T. (2008). In situ formation of platinum nanoparticles in Nafion recast film for catalyst-incorporated ion-exchange membrane in fuel cell applications. J. Membr. Sci..

[37] Donnadio A., D’Amato R., Marmottini F., Panzetta G., Pica M., Battocchio C., Capitani D., Ziarelli F., Casciola M. (2019). On the evolution of proton conductivity of Aquivion membranes loaded with CeO2 based nanofillers: Effect of temperature and relative humidity. J. Membr. Sci..

[38] Weissbach T., Peckham T.J., Holdcroft S. (2016). CeO_2_, ZrO_2_ and YSZ as mitigating additives against degradation of proton exchange membranes by free radicals. J. Membr. Sci..

[39] Radice S., Oldani C., Merlo L., Rocchia M. (2013). Aquivion^®^ PerfluoroSulfonic Acid ionomer membranes: A micro-Raman spectroscopic study of ageing. Polym. Degrad. Stab..

[40] Danilczuk M., Schlick S., Coms F.D. (2009). Cerium(III) as a Stabilizer of Perfluorinated Membranes Used in Fuel Cells: In Situ Detection of Early Events in the ESR Resonator. Macromolecules.

[41] Prabhakaran V., Arges C.G., Ramani V. (2012). Investigation of polymer electrolyte membrane chemical degradation and degradation mitigation using in situ fluorescence spectroscopy. Proc. Natl. Acad. Sci. USA.

[42] Xu K., Pei S., Zhang W., Han Z., Liu G., Xu X., Ma J., Zhang Y., Liu F., Zhang Y. (2022). Chemical stability of proton exchange membranes synergistically promoted by organic antioxidant and inorganic radical scavengers. J. Membr. Sci..

[43] Gubler L., Koppenol W.H. (2011). Kinetic Simulation of the Chemical Stabilization Mechanism in Fuel Cell Membranes Using Cerium and Manganese Redox Couples. J. Electrochem. Soc..

[44] Siracusano S., Oldani C., Navarra M.A., Tonella S., Mazzapioda L., Briguglio N., Aricò A.S. (2019). Chemically stabilised extruded and recast short side chain Aquivion^®^ proton exchange membranes for high current density operation in water electrolysis. J. Membr. Sci..

[45] Grot W. (2007). Fluorinated Ionomers.

[46] D’Urso C., Oldani C., Baglio V., Merlo L., Aricò A.S. (2014). Towards fuel cell membranes with improved lifetime: Aquivion^®^ Perfluorosulfonic Acid membranes containing immobilized radical scavengers. J. Power Sources.

[47] Pantò F., Siracusano S., Briguglio N., Aricò A.S. (2020). Durability of a recombination catalyst-based membrane-electrode assembly for electrolysis operation at high current density. Appl. Energy.

[48] Siracusano S., Van Dijk N., Payne-Johnson E., Baglio V., Aricò A.S. (2015). Nanosized IrOx and IrRuOx electrocatalysts for the O_2_ evolution reaction in PEM water electrolysers. Appl. Catal. B Environ..

[49] Siracusano S., Stassi A., Baglio V., Aricò A.S., Capitanio F., Tavares A.C. (2009). Investigation of carbon-supported Pt and PtCo catalysts for oxygen reduction in direct methanol fuel cells. Electrochim. Acta.

[50] Skulimowska A., Dupont M., Zaton M., Sunde S., Merlo L., Jones D.J., Rozière J. (2014). Proton exchange membrane water electrolysis with short-side-chain Aquivion^®^ membrane and IrO2 anode catalyst. Int. J. Hydrog. Energy.

[51] Barsoukov E., Ross Macdonald J. (2018). Impedance Spectroscopy: Theory, Experiment, and Applications.

[52] Carmo M., Fritz D.L., Mergel J., Stolten D. (2013). A comprehensive review on PEM water electrolysis. Int. J. Hydrog. Energy.

[53] Aricò A.S., Siracusano S., Briguglio N., Baglio V., Di Blasi A., Antonucci V. (2013). Polymer electrolyte membrane water electrolysis: Status of technologies and potential applications in combination with renewable power sources. J. Appl. Electrochem..

[54] Yukesh Kannah R., Kavitha S., Preethi, Parthiba Karthikeyan O., Kumar G., Dai-Viet N.V., Rajesh Banu J. (2021). Techno-economic assessment of various hydrogen production methods—A review. Bioresour. Technol..

[55] Proost J. (2019). State-of-the art CAPEX data for water electrolysers, and their impact on renewable hydrogen price settings. Int. J. Hydrog. Energy.

[56] Briguglio N., Siracusano S., Bonura G., Sebastián D., Aricò A.S. (2019). Flammability reduction in a pressurised water electrolyser based on a thin polymer electrolyte membrane through a Pt-alloy catalytic approach. Appl. Catal. B Environ..

[57] Cheikhravat H., Chaumeix N., Bentaib A., Paillard C.E. (2012). Flammability Limits of Hydrogen-Air Mixtures. Nucl. Technol..

